# Horizontal distribution of marine microbial communities in the North Pacific Subtropical Front

**DOI:** 10.3389/fmicb.2024.1455196

**Published:** 2024-12-24

**Authors:** Eva Lopes, Miguel Semedo, Maria Paola Tomasino, Renato Mendes, João Borges de Sousa, Catarina Magalhães

**Affiliations:** ^1^Faculty of Sciences, University of Porto, Porto, Portugal; ^2^Interdisciplinary Centre of Marine and Environmental Research (CIIMAR), University of Porto, Matosinhos, Portugal; ^3^+ATLANTIC CoLAB, Lisbon, Portugal; ^4^Underwater Systems and Technology Laboratory (LSTS), LAETA, Faculty of Engineering, University of Porto, Porto, Portugal

**Keywords:** North Pacific Subtropical Front, prokaryotes, eukaryotes, longitude, horizontal distribution

## Abstract

Microbial communities are crucial for important ecosystem functions in the open ocean, such as primary production and nutrient cycling. However, few studies have addressed the distribution of microplankton communities in the remote oligotrophic region of the Pacific Ocean. Moreover, the biogeochemical and physical drivers of microbial community structure are not fully understood in these areas. This research aims to investigate the patterns of prokaryotic and protists communities’ distribution in the North Pacific Subtropical Front (NPSF). The NPSF is a vast oligotrophic region with layered surface water and strong ocean currents. Despite its considerable size, its community distribution and function are poorly studied. We used a 16S and 18S rRNA gene sequencing approach to identify and characterize the water column microbial communities at two depths, the surface (3–5 m) and the deep chlorophyll maximum (DCM, 108–130 m). We aimed to elucidate the horizontal distribution patterns of these communities and to dissect the factors intricately shaping their distribution in the NPSF. Results showed that the community structure of both prokaryotes and protists was significantly influenced by depth, temperature, and longitude. Regarding alpha diversity, both communities presented a higher diversity at the surface. The prokaryotes also demonstrated to have a higher diversity in samples placed further east. The prokaryotes were dominated by Proteobacteria and Cyanobacteria, and the eukaryotic communities were dominated by Syndiniales. Combining biological and hydrographic data analysis showed the influence of vertical currents near the frontal jet in shaping the vertical distribution of both prokaryotic and protist communities. Even though most studies do not consider anomalies that emerge at each depth, these occurrences are capable of having a strong impact and influence on community structure. This study marks a significant advance in unraveling the intricate community structure and distribution dynamics of marine microbial communities within the North Pacific Ocean.

## 1 Introduction

The ocean is dominated by microbial life, prokaryotes, and eukaryotes, regarding abundance, diversity, and metabolic activity (Azam and Malfatti, 2007). Prokaryotic communities influence the shaping of the ecosystems, contributing to the elements’ cycles and the ocean’s energy flow (Ducklow, 2000; Kirchman, 2016; Steinberg and Landry, 2017). The protists communities also have a critical role in the ecosystem. These organisms have several trophic functions, acting as primary producers, consumers, parasites, and decomposers (Sherr and Sherr, 2002; Guillou et al., 2008; Davidson et al., 2010; Caron et al., 2012). Knowing these communities’ composition, how they are distributed, and what influences their distribution is especially important in the context of ocean fronts, since these communities represent the base of the food webs (Belkin et al., 2009).

Despite microbial communities being fundamental to the functioning of the ocean, the factors that influence these communities’ distribution are still poorly known, especially in remote areas such as the oligotrophic open ocean (Galand et al., 2010). The characteristics of the environment where the microbes inhabit have a great influence on community diversity and activity (Dang et al., 2010; Brockett et al., 2012). For a long time, prokaryotic and protists communities were described as stratified with depth (Giovannoni et al., 1996). Until recently, depth was considered the main reason for the differences found between marine microbial community structures in the water column (DeLong et al., 2006; Pham et al., 2008). However, several studies demonstrated that other factors, biotic and abiotic, can influence the depth profile of communities’ distribution. These factors include temperature, mixing in the water column, light availability, latitude, nutrient availability, water mass, inter-species interactions and competition, and predation (Nixon et al., 1995; Field et al., 1997; Karner et al., 2001; Matz and Jürgens, 2003; Giovannoni and Stingl, 2005; Hooper et al., 2005; Pernthaler, 2005; Howarth and Marino, 2006; Pommier et al., 2007; Agogué et al., 2008). However, it is fundamental to notice that there are connections between these factors, and they can overlap (Fu et al., 2019).

Spatial factors play an important role in shaping the distribution of prokaryotic and protists communities (Shurin et al., 2009). Ocean currents, water masses, and up/down-welling processes are factors that influence the horizontal distribution of these communities (Agogué et al., 2011; Bergen et al., 2015; Sunagawa et al., 2015). These elements create both a geographical influence and a distance-related pattern, indicating how the composition of communities changes with the distance that separates them (Nemergut et al., 2013; Wilkins et al., 2013; Lé Ne Morlon et al., 2008). Analysis in the Arctic and North Atlantic revealed that prokaryotic communities exhibited similarity across extensive distances within the same water mass but displayed distinctiveness between various water masses, even over relatively short distances (e.g., de Sousa et al., 2019). This can be caused by the horizontal transport of these microorganisms through ocean currents (Galand et al., 2010; Agogué et al., 2011; Wilkins et al., 2013). The up-and-down-welling currents are capable of vertically mixing the seawater and these microorganisms. Also, horizontal currents can mix the seawater at a single depth. Thus, a distance-decay relationship exists across the water column and the horizontal distance in seawater. These geographical patterns are also correlated with depth (Milici et al., 2016). Nevertheless, it is still unknown what differences exist between the patterns of distance-decay relationship across the water column and horizontal distance, as well as the factors driving these differences (Li Y. et al., 2018). Furthermore, the comparative analysis of these patterns across microbial community fractions, such as prokaryotic and eukaryotic, is rarely conducted. Considering the inherent distinctions in body composition, trophic level, and dispersal capacity among these microorganisms (Gong et al., 2023), such investigations hold significant relevance for advancing our understanding of how the microbial communities are distributed in the ocean. An ocean front can be defined as a narrow area of intensified horizontal gradients of water physicochemical properties, such as temperature, salinity, and nutrients, which divides extensive areas according to their water masses or according to the water column stratification, i.e., their vertical structure (Belkin et al., 2009). Fronts have critical roles in marine ecosystems, such as increasing productivity, and are part of the migratory routes for several species (Belkin et al., 2009). The North Pacific Subtropical Front (NPSF) is described as one of the biggest oligotrophic areas on the planet, presents stratified surface waters, and displays the largest oceanic currents (Polovina et al., 2008; Spalding et al., 2012; Tseng et al., 2016; Li Y. Y. et al., 2018). Even though this front presents a relevant geographic area, it is rarely studied regarding its communities’ distribution and functionality, despite a few recent advances in the N cycling-mediated microbial communities (Tseng et al., 2016; Karl and Church, 2017; Kavanaugh et al., 2018; Li Y. Y. et al., 2018; Semedo et al., 2021).

In this study, we investigate the patterns of horizontal distribution of prokaryotic and protist communities across the NPSF, to understand how the physical forces and biogeochemical gradients that characterize this remote region influence these planktonic communities.

## 2 Materials and methods

### 2.1 Site description and water sampling

**FIGURE 1:**
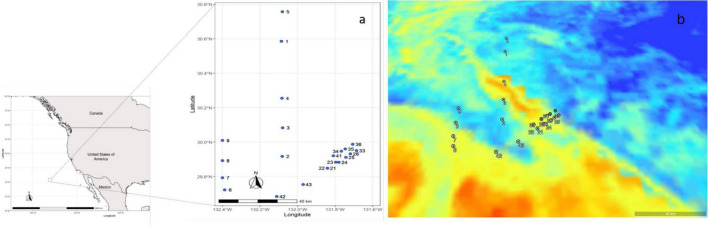
Map of the sampling area and sampling sites **(a)** sample location identified by cast number **(b)** satellite image of the study area, where the image corresponds to surface samples. Blue on the map represents the polar water mass, and red represents the subtropical water mass.

Seawater samples were collected in four transects, along a latitudinal and longitudinal gradient in the North Pacific Subtropical Front (NPSF), 1,000 nautical miles off the Southern California coast (Figure 1). Through 22 Casts, a total of 34 samples were collected with the Rosette multi-sampler, between the 1st and 14th of June 2018, within a depth range of 3 to 130 m (Table 1). Seawater samples of 3.75 L were filtered with a Sterivex filter (0.2 μm pore size). The collection filters were stored onboard at −80°C and transported in dry ice to CIIMAR for later DNA extraction. Samples were classified according to their depth and *in situ* chlorophyll concentrations, resulting in two different groups of samples: Surface (3–5 m; *n* = 22) and deep chlorophyll maximum -DCM (108–130 m; *n* = 12). The DCM depths observed in this study were similar to the DCM depths previously observed in the Pacific Ocean (Letelier et al., 2004; Sauzède et al., 2018). Additionally, three different types of water masses were identified: Polar (*n* = 13), Front (*n* = 12) and Subtropical (*n* = 9). The salinity values of the Polar water samples varied from 34.07 to 34.29, the Front samples varied from 34.34 to 34.66, and the Subtropical water samples varied from 34.7 to 34.96. The salinity values and water mass classification matched Aksenov et al. (2010), Cao et al. (2020), and Woo and Pattiaratchi (2008). Detailed information about sampling and filtration methodologies are available in Semedo et al. (2021).

**TABLE 1:** Geospatial description of samples used in this study.

Depth layer	Transect	Sample ID	Water mass	Cast	Date	Time	Latitude	Longitude	Temperature (°C)	Salinity PSU	Depth (m)
Surface (3–5 m)	A	4A	Polar	1	01/06/2018	6:17	30.586	−132.08606	18.64	34.21	5
		8A	Front	2	01/06/2018	14:00	29.9179	−132.0818	19.11	34.66	5
		12A	Subtropical	3	02/06/2018	5:20	30.084	−132.08	19.46	34.78	5
		16A	Subtropical	4	02/06/2018	9:25	30.255	−132.083	19.50	34.80	5
		20A	Polar	5	02/06/2018	15:08	30.7575	−132.081	18.72	34.15	5
	B	21A	Subtropical	6	06/06/2018	7:10	29.7239	−132.3879	19.6	34.73	5
		22A	Front	7	06/06/2018	7:42	29.7938	−132.4	19.3	34.55	5
		23A	Front	8	06/06/2018	8:26	29.893	−132.4	18.8	34.34	5
		24A	Polar	9	06/06/2018	9:17	30.0104	−132.4	18.45	34.18	5
	C	53A	Subtropical	33	11/06/2018	22:06	29.87848	−131.8647	19.49	34.81	3
		55A	Front	34	11/06/2018	23:29	29.94787	−131.76732	19.08	34.39	3
		57A	Polar	35	12/06/2018	0:28	29.96118	−131.74621	18.76	34.20	3
		59A	Polar	36	12/06/2018	1:50	29.98813	−131.70628	18.58	34.08	3
		69A	Front	41	13/06/2018	2:24	29.92122	−131.80977	19.52	34.39	3
		71A	Subtropical	42	14/06/2018	2:45	29.68509	−132.11068	20.01	34.96	3
		73A	Front	43	14/06/2018	4:11	29.75496	−131.97031	19.05	34.42	3
	D	40A	Subtropical	21	09/06/2018	23:27	29.84946	−131.84066	20.12	34.74	3
		41A	Subtropical	22	09/06/2018	23:27	29.84946	−131.84066	20.14	34.76	5
		42A	Subtropical	23	10/06/2018	0:15	29.88433	−131.7968	20.17	34.88	5
		43A	Subtropical	24	10/06/2018	1:16	29.88352	−131.78023	20.11	34.7	5
		44A	Polar	25	10/06/2018	1:35	29.91247	−131.74263	19.65	34.28	5
		45A	Polar	26	10/06/2018	2:00	29.93268	−131.7192	19.41	34.13	5
DCM (108–130m)	A	3A	Front	1	01/06/2018	6:17	30.586	−132.08606	16.59	34.40	120
		7A	Polar	2	01/06/2018	14:00	29.9179	−132.0818	15.61	34.18	110
		11A	Front	3	02/06/2018	5:20	30.084	−132.08	16.52	34.36	108
		15A	Polar	4	02/06/2018	9:25	30.255	−132.083	16.22	34.29	122
		18A	Polar	5	02/06/2018	15:08	30.7575	−132.081	15.76	34.07	130
	C	52A	Front	33	10/06/2018	2:35	29.9514	−131.68668	16.71	34.40	123
		54A	Polar	34	11/06/2018	23:29	29.94787	−131.76732	16.45	34.28	115
		56A	Polar	35	12/06/2018	0:28	29.96118	−131.74621	16.63	34.28	115
		58A	Polar	36	12/06/2018	1:50	29.98813	−131.70628	17.15	34.29	115
		68A	Front	41	13/06/2018	2:24	29.92122	−131.80977	16.62	34.44	125
		70A	Front	42	14/06/2018	2:45	29.68509	−132.11068	17.13	34.51	107
		72A	Front	43	14/06/2018	4:11	29.75496	−131.97031	17.56	34.61	107

### 2.2 Physiochemical parameters

Physicochemical properties of the collected water samples were obtained *in situ* with a Seabird SBE 9 Plus CTD (conductivity-temperature-depth) profiler, deployed with the Rosette. Conductivity (mS/cm), temperature (°C), depth (m), salinity (PSU), oxygen (ml/L), turbidity (NTU), and fluorescence (mg/m3) were measured simultaneously in each cast and the complete results from the CTD dataset are publicly available in PANGAEA.^1^ In addition, water samples were collected to quantify the inorganic nitrogen, i.e., ammonium (NH_4_^+^), nitrite (NO_2_^–^), and nitrate (NO_3_^–^), as well as silica (SiO_2_) and phosphate (PO_4_^3–^), at the stations and depths where microplankton samples were collected. These samples were also stored onboard at −80°C. Upon arrival to shore, nutrient samples were transported in dry ice to the Technical University of Cartagena, Spain, to be analyzed using a SEAL AA3-HD continuous flow autoanalyzer according to the previously described methodology (Strickland and Parsons, 1972; Field et al., 1997). A subset of these data was previously published to investigate nitrogen cycling dynamics (Semedo et al., 2021).

### 2.3 DNA extraction and amplicon sequencing

As previously described (Semedo et al., 2021), planktonic DNA was extracted from the Sterivex filters using the DNeasy^®^ PowerWater^®^ Sterivex DNA Isolation Kit protocol (Qiagen), following the manufacturer’s instructions. The 16S rRNA gene was amplified with the degenerate primer pair 515YF (5′–GTGYCAGCMGCCGCGGTAA–3′) and Y926R-jed (5′–CCGYCAATTYMTTTRAGTTT–3′), targeting the hypervariable V4-V5 region (Apprill et al., 2015; Caporaso et al., 2011; Parada et al., 2016). The 18S rRNA gene was amplified with the primer set described in Stoeck et al. (2010), TAReuk454FWD1 (5′–CCAGCASCYGCGGTAATTCC–3′) and TAReukREV3_modified (5′–ACTTTCGTTCTTGATYRATGA–3′). The initial PCR, using reaction, included 12.5 ng of template DNA in a total volume of 25 μL, using DreamTaq PCR Master Mixes (2X). The PCR protocol involved a 3 min denaturation step, followed by 25 cycles of 98°C for 20 s, 60°C for 30 s, and 72°C for 30 s, and, finally, an extension at 72°C for 5 min. Negative controls without templates were included in all PCR reactions. Lastly, PCR products were one-step purified and normalized using a SequalPrep 96-well plate kit (ThermoFisher Scientific, Waltham, USA), pooled, and pair-end sequenced in the Illumina MiSeq^®^ sequencer using 2 × 300 bp with the V3 chemistry, according to manufacturer instructions (Illumina, San Diego, CA, USA) at Genoinseq (Cantanhede, Portugal). The results from the 16S and 18S rRNA gene sequencing are publicly available in the ENA-EMBL archive with the project accession number PRJEB32783.

### 2.4 Bioinformatic analysis

The raw FASTQ files obtained with Illumina MiSeq sequencing were trimmed for primer removal using “cutadapt” v.1.16 and imported into R (version 3.6.1) using “DADA2” package v.1.14.1 (Callahan et al., 2016). Sample filtering, trimming, error rates learning, dereplication, and amplicon sequence variant (ASV) inference were performed with default settings. Chimeras were removed with the *removeBimeraDenovo* function using the method “consensus.” Taxonomy was assigned with the native implementation of the naive Bayesian classifier and a DADA2-formatted reference database for the SILVA v132 database, for 16S taxonomical analysis (Quast et al., 2013). As for the 18S taxonomic analysis, the PR^2^ v4.13.0 database was used (Guillou et al., 2013). For the 16S analysis, these pre-processing steps resulted in 6433 ASVs found, with a median number of 38,414 reads per sample, corresponding to 47.18% of the initial number of the sequences (Supplementary Table 1). For the 18S analysis, these pre-processing steps resulted in 10,330 ASVs found, with a median number of 44,098 reads per sample, corresponding to 65.13% of the initial number of sequences (Supplementary Table 2). Taxonomy filtering was performed by removing eukaryotic, mitochondrial, and chloroplast sequences from the 16S database. As for the 18S database, non-specific lineages such as “Metazoa,” “Fungi,” “Streptophyta,” and “Ulvophyceae” were excluded. Relative abundances of each ASV per sample were calculated in the filtered dataset by dividing the absolute abundance (counts) of each ASV by the sum of counts of all ASVs.

To estimate species richness, total number of ASVs found per sample, and species diversity of the microbial communities, observed metrics and Shannon indexes were calculated, respectively. β-diversity among these communities was evaluated using the Bray-Curtis dissimilarity calculator, using the Vegan package in R (v. 2.5-7; Oksanen et al., 2020). Significant effects of physicochemical parameters and geographic coordinates in communities dissimilarities were tested by multivariate permutational ANOVA (PERMANOVA) using the Adonis function of the vegan package in R (Oksanen et al., 2020). To normalize the diversity estimates, the sequence dataset was randomly subsampled to the lowest number of sequences (*n* = 17,032 sequences per sample for 16S and *n* = 30,248 sequences per sample for 18S). These estimates were calculated using the phyloseq package in R (McMurdie and Holmes, 2013).

### 2.5 Statistical analysis

Differences in the α-diversity between the two depth groups (surface and DCM), for both 16S and 18S, were analyzed using the *t*-test. A hierarchical cluster was performed to represent the β-diversity of the prokaryotic and eukaryotic communities, based on dissimilarity among samples, using the vegan R package (v. 2.5-7; Oksanen et al., 2020). Significant relationships were considered at α−0.05, and *p*-values were adjusted to account for multiple comparisons, using the Benjamini-Hochberg (BH) method (Benjamini and Hochberg, 1995). Spearman’s rank correlation coefficients were employed to assess the correlations between the genera and the geographic coordinates and the physicochemical parameters. To account for multiple comparisons, *p*-values were adjusted accordingly, ensuring the validity of the statistical results, with the same parameters as before. Low abundance ASVs (that do not appear more than two times in at least four samples) were excluded from this analysis to avoid low degrees of freedom, as previously performed (Semedo et al., 2021). Correlations were obtained on a centered log-ratio transformed ASV table (Gloor et al., 2017). Distance decay relationship (this is, the effect of geographical distance on community similarity), at both depths, was determined using untransformed values of geographic distance against microbial community similarity Bray–Curtis distance for ASVs for all taxonomy ranks. Geographical distances between samples were calculated using the distm function with the distGeo formula, using the geosphere R package (v. 1.5-1.4; Hijmans et al., 2019). Statistical analyses were conducted in the R environment (version 3.2.2. Copyright 2015, the R Foundation for Statistical Computing). Most plots were obtained with base R and the ggplot2 R package.

## 3 Results

### 3.1 Environmental characteristics

The physicochemical parameters and nutrient concentrations measured at the different stations and depths are shown at Table 2. No clear latitudinal or longitudinal trends were observed for any of the physicochemical and nutrient parameters measured in the four transects. The temperature decreased with depth, from a mean of 19.35°C at the surface to 15.68°C at DCM. Salinity values were relatively constant with depth (surface mean = 34.51; DCM mean = 34.34), but were used to identify the different water masses present in the area (Table 1 and Material and Methods). Turbidity values were also relatively stable (surface mean = 0.06 NTU; DCM mean = 0.05 NTU). However, there was a pronounced increase in the fluorescence values with depth, from a mean of 0.07 mg/m3 at the surface to 0.55 mg/m3 at DCM, as expected.

**TABLE 2:** Physicochemical parameters at surface and DCM.

Cast	Depth	Sample ID	Temperature (°C)	Salinity	O2 (ml/L)	Turbidity (NTU)	Fluorescence (mg/m^3^)	Silica (μmol/L)	Phosphates (μmol/L)	Nitrites (μmol/L)	Nitrates (μmol/L)	Ammonium (μmol/L)
1	DCM	3A	16.59	34.40	5.22	0.05	0.63	1.68	0.25	0.0150	0.0150	0.04
	Surface	4A	18.64	34.21	5.26	0.06	−0.02	1.18	0.11	0.0150	0.0150	0.04
2	DCM	7A	15.61	34.18	5.21	0.06	0.56	2.15	0.36	0.0934	0.0150	0.04
	Surface	8A	19.11	34.66	5.21	0.06	−0.02	1.08	0.18	0.0150	0.0150	0.04
3	DCM	11A	16.52	34.36	5.25	0.06	0.66	1.73	0.25	0.0150	0.0150	0.04
	Surface	12A	19.46	34.78	5.16	0.08	−0.03	0.95	0.16	0.0150	0.0150	0.04
4	DCM	15A	16.22	34.29	5.26	0.06	0.67	2.01	0.29	0.0812	0.0150	0.04
	Surface	16A	19.50	34.80	5.16	0.06	−0.02	1.17	0.15	0.0107	0.0150	0.04
5	DCM	18A	15.76	34.07	5.33	0.07	0.56	1.23	0.12	0.0276	0.0150	0.04
	Surface	20A	18.72	34.15	5.25	0.06	0.00	1.17	0.15	0.0064	0.0150	0.04
6	Surface	21A	19.6	34.73	NA^1^	NA	NA	1.36	0.14	0.0150	0.0150	0.04
7	Surface	22A	19.3	34.55	NA	NA	NA	1.60	0.14	0.0150	0.0150	0.04
8	Surface	23A	18.8	34.34	NA	NA	NA	2.01	0.19	0.0150	2.0886	0.04
9	Surface	24A	18.45	34.18	NA	NA	NA	2.60	1.46	0.0403	0.0150	0.04
21	Surface	40A	20.12	34.74	NA	NA	NA	1.26	0.37	0.0150	0.0150	0.04
22	Surface	41A	20.14	34.76	NA	NA	NA	1.26	0.37	0.0150	0.0150	0.04
23	Surface	42A	20.17	34.88	NA	NA	NA	1.10	0.32	0.0150	0.0150	0.04
24	Surface	43A	20.11	34.7	NA	NA	NA	1.05	0.32	0.0088	0.0150	0.04
25	Surface	44A	19.65	34.28	NA	NA	NA	0.98	0.31	0.0150	0.0150	0.04
26	Surface	45A	19.41	34.13	NA	NA	NA	1.10	0.29	0.0150	0.0150	0.04
33	DCM	52A	16.71	34.40	NA	NA	NA	1.05	0.32	0.0150	0.0150	0.04
	Surface	53A	19.49	34.81	NA	NA	NA	0.98	0.25	0.0552	0.0150	0.04
34	DCM	54A	16.44	34.28	NA	NA	NA	2.06	0.31	0.0036	0.0150	0.04
	Surface	55A	19.08	34.39	NA	NA	NA	1.13	0.25	0.0150	0.0150	0.04
35	DCM	56A	16.63	34.28	NA	NA	NA	1.45	0.30	0.0150	0.0150	0.04
	Surface	57A	18.76	34.20	NA	NA	NA	1.26	0.26	0.0150	0.0150	0.04
36	DCM	58A	17.15	34.29	NA	NA	NA	1.05	0.33	0.0150	0.0150	0.04
	Surface	59A	18.58	34.08	NA	NA	NA	1.25	0.41	0.0150	0.0150	0.04
41	DCM	68A	16.62	34.44	5.04	0.04	0.66	1.70	0.38	0.0150	0.0150	0.04
	Surface	69A	19.52	34.39	5.04	0.04	0.66	1.40	0.30	0.0150	0.0150	0.04
42	DCM	70A	17.13	34.51	5.15	0.03	0.49	1.56	0.34	0.0150	0.0150	0.04
	Surface	71A	20.01	34.96	5.09	0.03	0.00	1.20	0.19	0.0150	0.0150	0.04
43	DCM	72A	17.56	34.61	5.26	0.03	0.14	2.10	0.43	0.0150	0.0150	0.04
	Surface	73A	19.05	34.42	4.84	0.06	−0.02	1.10	0.22	0.0150	0.0150	0.04

^1^NA: NA (not available) correspond to samples that were not collected with the Rosette system, as the CTD was not deployed at these stations.

Regarding the concentration of nutrients, phosphate (PO4^3–^) was almost constant at both depths (surface mean = 0.29 μmol P/L; DCM mean = 0.31 μmol P/L). As well as, the concentration of silica (SiO_2_), from a mean of 1.28 μmol/L at Surface to a mean of 1.65 μmol/L at DCM. Despite having a concentration peak at DCM, nitrite (NO_2_^–^) values were relatively low at both depths. Nitrate (NO_3_^–^) concentrations presented values below the limit of quantification in most of the samples (< 0.0150 μM), with one exception at the surface where it reached 2.09 μM. Dissolved NH_4_^+^ concentrations at both depths were below the limit of quantification (< 0.04 μM). Regarding the spatial differences in nutrient concentration samples, no specific pattern is discernible. With respect to the samples located further east, sample 24A (Depth: Surface; Water Mass: Polar) exhibits elevated levels of silica, phosphates, and nitrites. Conversely, sample 23A (Depth: Surface; Water Mass: Front) demonstrates a notable peak in nitrate concentrations. Samples 21A (Depth: Surface; Water Mass: Subtropical) and 22A (Depth: Surface; Water Mass: Front) display the lowest values for these nutrients across the analyzed dataset.

### 3.2 Microbial communities structure and diversity

**FIGURE 2:**
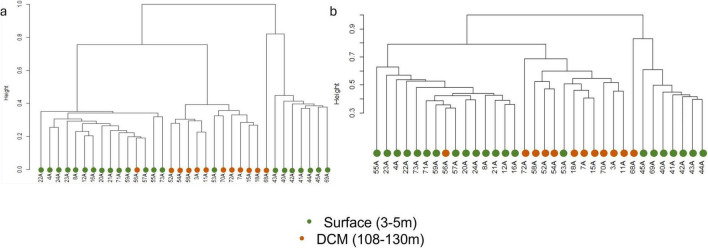
Hierarchical cluster of prokaryotic **(a)** and eukaryotic **(b)** communities.

Beta diversity analysis showed that the prokaryotic communities were divided into three main clusters (Figure 2a), regarding their community structure dissimilarity. Samples from the surface were split into two clusters and samples from DCM were included in the same cluster. However, there were some exceptions: samples 56A and 68A, collected at the DCM, were present in the surface clusters, while sample 53A, collected at the surface, was included in the DCM cluster. When considering the relationship between community structure and the environmental variables, prokaryotic β-diversity was significantly influenced by depth, temperature, and longitude (PERMANOVA, *p* < 0.05) (Supplementary Table 3A). The protists communities presented a similar pattern with depth when compared with the prokaryotic communities (Figure 2b), with two surface and one DCM cluster. Eukaryotic β-diversity was also significantly influenced by depth, temperature, and longitude (PERMANOVA, *p* < 0.05) (Supplementary Table 3B). The beta diversity of both communities demonstrated that there were two distinct surface groups. The cluster constituted with the surface samples 40A, 41A, 42A, 43A, 44A, 45A, and 69A presented the highest values of temperature and phosphates (Supplementary Table 4).

**FIGURE 3:**
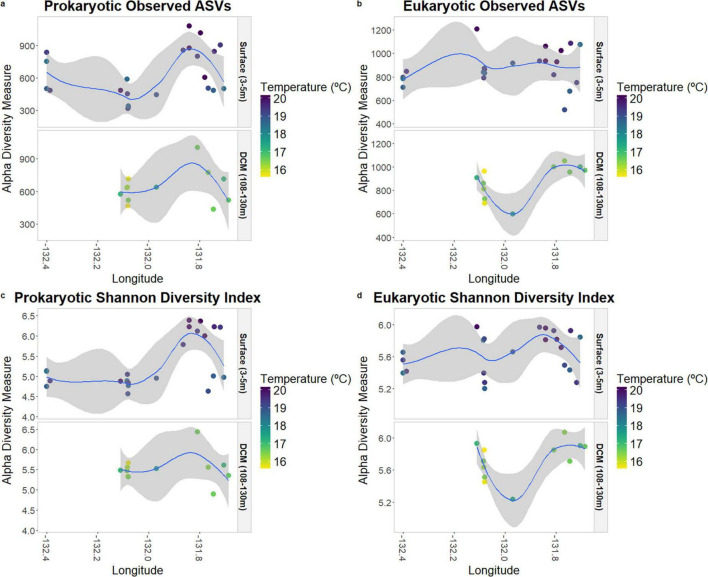
Species richness and alpha-diversity of prokaryotic **(a,c)** and eukaryotic **(b,d)** communities at surface and DCM.

Species richness (“Observed ASVs”) and species diversity (“Shannon Index”) are displayed in Figure 3, according to depth, temperature, and longitude. In the prokaryotic communities, it is possible to observe that samples placed further east present higher values of diversity, with the Shannon index significantly correlated with longitude (*p*-value = 0.02; Supplementary Table 4), especially at the surface. Regarding temperature, the samples located further east also presented higher values of temperature.

Within the protists community, there was no significant correlation between species richness and diversity concerning longitude (Supplementary Table 4), unlike the pattern observed among the prokaryotes.

### 3.3 Community composition

**FIGURE 4:**
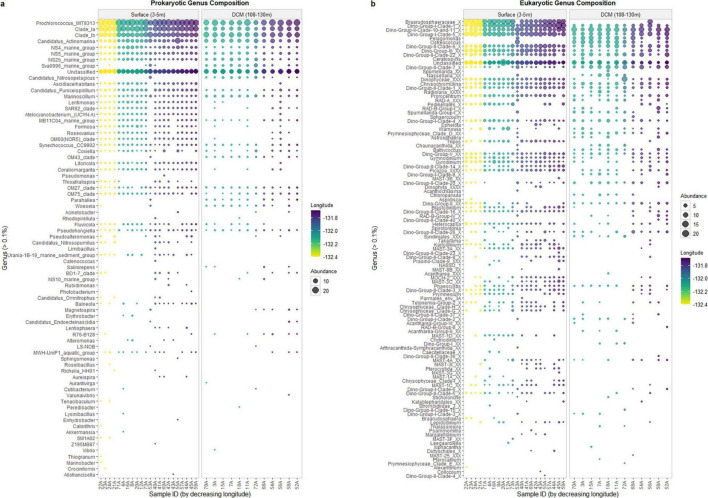
Bubble plot cluster of relative abundance of prokaryotic **(a)** and eukaryotic **(b)** communities at genus level.

The prokaryotic community was mainly composed of Proteobacteria and Cyanobacteria (Supplementary Figure 1). As for the protists’ community, Alveolata presented the highest abundance (Supplementary Figure 2). The relative abundances of the most abundant genera of the prokaryotic and protists communities are shown in Figure 4.

**FIGURE 5:**
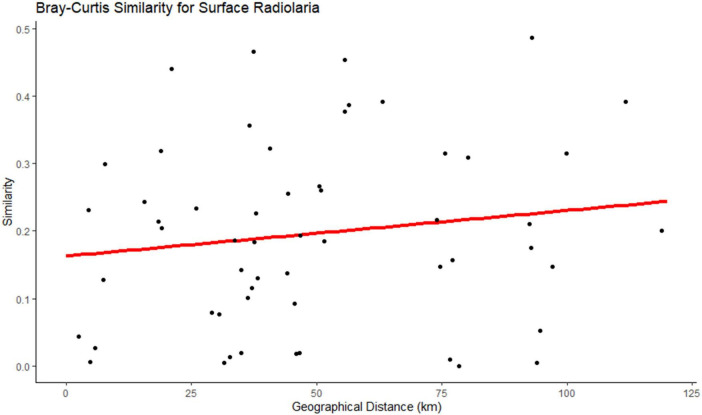
Bray-Curtis similarity showing the distance-decay relationship in surface Radiolaria.

Considering the prokaryotic community, it is possible to detect differences in the communities’ composition within the two depths (surface and DCM), at the genus level (Figure 4a). The main differences in the communities’ composition are detected in samples 53A (surface) and sample 56A (DCM). The surface sample 53A presents a composition similar to DCM samples, and the DCM sample 56A showed a community similar to surface samples, which is consistent with the hierarchical clustering observed (Figure 2). Genera such as *Ascidiaceihabitans*, *Lentimonas*, *Atelocyanobacterium (UCYN-A)*, *OM60(NOR5) Clade*, and *Litoricola* are exclusively observed in the sample 56A among all DCM samples, while being observed at all surface samples, except for sample 53A. On the other hand, the genus *Sva0996 Marine Group* can only be found in sample 53A among all surface samples, whereas it is observed in all DCM samples, except for sample 56A. To determine how the microbial genera related to the environmental characteristics, a Spearman’s rank correlation coefficient between the genera’s relative abundance, environmental parameters (temperature and salinity), and geographic coordinates was calculated. *Litoricola* revealed a significant positive relationship with temperature (ρ > | 0.7|, *p*-value < 0.05; Supplementary Figure 3). This genus is found in all surface samples, except sample 53A, and is observed in sample 56A. Conversely, genera present in all DCM samples, except for sample 56A, also presented a significant Spearman relationship (*p* < 0.05) with temperature. These genera are *Sva0996 Marine Group*, *Ascidiaceihabitans*, *Lentimonas*, *Atelocyanobacterium (UCYN-A)* and *OM60(NOR5) Clade*. Through this analysis, it is possible to support the results of α-diversity of the communities, regarding the longitude effect: samples located further east present a higher diversity and species richness in comparison with the samples located further west, at both depths. In these samples, it is possible to find more low-abundant genera. Such as the genera *Aureispira* and *Magnetospira*.

As for the protists community, it was also possible to detect a composition difference in samples 53A and 56A (Figure 4b). For instance, genera *Braarudosphaeraceae X* and *Tripos* are exclusively found in sample 56A, at DCM, while being commonly observed in surface samples, except for sample 53A. Contrarily, the genera *Pelagomonas*, and *Ostreococcus* are only observed in sample 53A, at the surface samples, and are observed in all DCM samples, except for sample 56A. A Spearman’s rank correlation coefficient between the genera relative abundance, environmental parameters (temperature and salinity), and geographic coordinates was also calculated. Regarding temperature, *Prymnesiophyceae Clade-D-XX* was the only genus that demonstrated a significant negative relationship with temperature (ρ > | 0.7|, *p*-value < 0.05; Supplementary Figure 4). The genera *Karlodinium*, *Strombidium-K*, *Rhizosolenia*, *Strombidiidae_L_X*, and *Tripos* revealed a significant positive relationship with temperature (ρ > | 0.7|, *p*-value < 0.05; Supplementary Figure 4). In addition, more significant Spearman relationships (*p*-value < 0.05) were also identified. The genera *Braarudosphaeraceae-X*, *Pelagomonas*, *Ostreococcus*, *Bathycoccus*, and *Blastodinium* were significantly related to temperature, and *Dino-Group-II-Clade-14-X* and *MOCH-2 XXX* presented a significant Spearman relationship (*p*-value < 0.05) with temperature and longitude.

### 3.4 Community similarity with geographic distance

A distance decay relationship (DDR) analysis was performed to observe the effect of geographic distance, in all taxonomic ranks, on the microbial community similarity at both depths, surface, and DCM. To avoid extremely underrepresented communities, we excluded the ASVs with less, or equal, than eight observations in each sample.

In the prokaryotic communities, the Bray-Curtis similarity showed that only nine genera (Table 3), at the surface, had a significant correlation with the horizontal geographic distance over the 118.92 Km scale investigated in this study. The slopes of the distance-decay relationship were steeper for the genera *Thioalkalispira* (−0.00443) and *Candidatus nitrosopumilus* (−0.00452) (Supplementary Figure 5). In contrast, no significant correlation with the horizontal geographic distance was found at DCM in the prokaryotic communities. In the protists communities (Table 4), the Bray-Curtis similarity showed that at both depths (surface *n* = 49 genera; DCM *n* = 10 genera) was possible to find a significant correlation with the horizontal geographic distance over a scale of 118.92km. The slopes of the distance-decay relationship were steeper for the genera *Pentapharsodinium* (−0.009) and *Syndiniales XXX* (−0.0082) at the surface, and at DCM the slopes were steeper for the genera *Dino_Group_II_Clade_55_X* (−0.0098) and *Prymnesiophyceae XXX* (−0.00511) (Supplementary Figure 5).

**TABLE 3:** Prokaryotic taxa with a significant relationship between Bray-Curtis similarity and geographic distance.

Depth	Classification	*p*-value	Model *R*^2^	Slope
Surface	*Formosa*	0.002149	0.1339	−0.00251
Surface	*Marinoscillum*	0.005719	0.07608	0.002014
Surface	NS4_marine_group	0.001688	0.07795	0.002401
Surface	NS5_marine_group	1.21E-05	0.1631	0.002741
Surface	Urania-1B-19_marine_sediment_group	0.03901	0.06116	−0.00228
Surface	*Ascidiaceihabitans*	0.04622	0.03596	0.001175
Surface	*Roseovarius*	0.01186	0.05246	0.002065
Surface	*Thioalkalispira*	0.03812	0.1447	−0.00443
Surface	Candidatus_Nitrosopumilus	0.04278	0.3818	−0.00452

**TABLE 4:** Eukaryotic taxa with a significant relationship between Bray-Curtis similarity and geographic distance.

Depth	Classification	*p*-value	Model *R*^2^	Slope
Surface	Chytriodinium	0.01897	0.4751	−0.01083
Surface	Dino_Group_II_Clade_10_and_11_X	0.04587	0.03176	−0.00114
Surface	Dino_Group_II_Clade_14_X	0.002565	0.07635	−0.00173
Surface	Dino_Group_II_Clade_15_X	2.86E-06	0.2771	−0.00411
Surface	Dino_Group_II_Clade_17_X	0.04288	0.1965	−0.00124
Surface	Dino_Group_II_Clade_26_X	0.03771	0.03464	−0.00108
Surface	Dino_Group_II_Clade_28_X	0.000826	0.3244	−0.00699
Surface	Dino_Group_II_Clade_5_X	0.01743	0.1921	−0.00139
Surface	Dino_Group_II_Clade_52_X	0.006663	0.1435	−0.00242
Surface	Dinophyta_XXXX	0.02353	0.05124	0.001602
Surface	Eutintinnus	0.01105	0.1621	−0.00253
Surface	Gyrodinium	0.000949	0.08465	−0.00129
Surface	Mesanophrys	0.003077	0.6408	−0.0089
Surface	OLIGO5_XX	0.03663	0.05096	−0.00148
Surface	*Pentapharsodinium*	0.04683	0.4533	−0.009
Surface	PHYLL_4_X	0.01045	0.09525	−0.00173
Surface	Strombidiida_D_XX	0.0446	0.1103	−0.00263
Surface	Strombidiidae_J_X	0.01648	0.1163	−0.00195
Surface	*Strombidiidae_L_X*	0.000667	0.1189	0.002118
Surface	Syndiniales_XXX	0.008621	0.5988	−0.0082
Surface	Tontoniidae_A_X	0.005787	0.4553	−0.00647
Surface	Warnowia	0.001164	0.1135	−0.00185
Surface	Apusomonadidae_Group_2B_XX	4.68E-05	0.5368	−0.00571
Surface	Chloroparvula	0.000609	0.1738	−0.00433
Surface	Halosphaera	0.01202	0.07914	−0.0017
Surface	Pterosperma	0.01549	0.1317	−0.00267
Surface	Algirosphaera	1.22E-05	0.3557	−0.00419
Surface	Phaeocystis	0.01881	0.04916	−0.00114
Surface	Prymnesium	0.02132	0.04767	−0.00104
Surface	Choanoflagellida_XX_Clade_3_X	0.03883	0.05496	−0.00166
Surface	Acanthometron	5.96E-08	0.3487	−0.00419
Surface	Lithomelissa	0.02559	0.08737	−0.00246
Surface	Micrometopion	0.004086	0.2511	−0.00693
Surface	Xiphacantha	0.01015	0.08827	−0.00214
Surface	Chaetoceros	0.03615	0.05121	0.001818
Surface	Chrysophyceae_Clade_I_X	0.009802	0.0591	−0.00177
Surface	Chrysophyceae_XXX	0.01106	0.1752	−0.0032
Surface	Dictyocha	0.002856	0.1084	−0.00238
Surface	Fragilariopsis	0.005042	0.3316	−0.00711
Surface	MAST_12D_XX	0.000577	0.4723	−0.00724
Surface	MAST_1A_XX	0.003791	0.07368	−0.0024
Surface	MAST_1D_XX	0.01313	0.04898	−0.00174
Surface	MAST_3A_XX	0.02204	0.0476	−0.00113
Surface	MAST_3I_XX	1.82E-06	0.1878	−0.00291
Surface	MAST_4A_XX	0.04603	0.03172	−0.00117
Surface	MAST_7C_XX	0.008745	0.07314	−0.0022
Surface	MAST_7D_XX	0.01555	0.2289	−0.00511
Surface	Pelagococcus	2.82E-05	0.8094	−0.00845
Surface	Triparma	1.67E-05	0.1948	−0.00327
DCM	Dino_Group_II_Clade_17_X	0.0242	0.1805	−0.00279
DCM	Dino_Group_II_Clade_24_X	0.008033	0.364	−0.00484
DCM	Dino_Group_II_Clade_37_X	0.01238	0.1339	−0.00348
DCM	Dino_Group_II_Clade_55_X	0.003534	0.5538	−0.0098
DCM	Dinophyta_XXXX	0.02771	0.2682	0.003449
DCM	Prymnesiophyceae_XXX	0.008052	0.3462	−0.00511
DCM	Prymnesium	0.0013	0.4457	−0.005
DCM	Spumellarida_XX	0.04233	0.1608	−0.0024
DCM	MAST_3E_XX	0.03536	0.09893	0.00245
DCM	Pedinellales_X	0.03559	0.117	−0.00285

## 4 Discussion

Ocean fronts are usually described as the transition zone between mixed and stratified waters and can act as an oceanographic barrier for the prokaryotic and eukaryotic communities (Baltar and Arístegui, 2017). The North Pacific Subtropical Front (NPSF) is vertically stratified and depth, being a proxy for light entrance, is generally referred to as the principal driver for microbial zonation (DeLong et al., 2006; Semedo et al., 2021). According to a study on rRNA gene data from the Tara oceans performed in similar depths (0–200 m), environmental parameters were the main drivers of communities’ distribution, especially the water temperature (Logares et al., 2020). Our findings further showed the substantial influence of water temperature, revealing distinct communities between the surface and DCM depths.

Temperature variations are commonly pointed to as the physicochemical gradient that influences the prokaryotes’ distribution the most (Yu et al., 2015). Besides temperature, salinity is another physicochemical parameter that has a strong influence on the water mass characteristics and on the communities that inhabit it. In our study, the salinity and temperature gradients across the study area were not very sharp, in comparison with other studied fronts (Pommier et al., 2007; Yu et al., 2015). Nevertheless, it was possible to detect the influence of longitude and depth (PERMANOVA, *p* < 0.05) in both prokaryotic and protists communities, leading to two distribution irregularities in each depth. These irregularities are evident as outliers in the distribution analysis. For example, sample 53A from the surface often contains microorganisms typically found in DCM samples, while the DCM sample frequently contains microorganisms typical of surface samples.

Ocean fronts are highly dynamic regions. These regions tend to be zones of convergence, which leads to the accumulation of organic material. In addition, intense sub-mesoscale instability is often associated with the margins of the main front, where this cruise took place. Here, a sub-mesoscale counter-clockwise spiral-like feature was observed, an ocean structure partially detached from the main front with extreme surface velocities in the order of 1 m/s (Pinto et al., 2022). The results in this location present strong instability associated with the structure. The scale of the jet also suggests a strong horizontal shear and a cross-frontal convergence, with strong vertical velocities, which implied a vertical movement of the water masses in the border of the structure.

Near the boundaries of the spiral-like feature, the rapid horizontal shift in the magnitude and speed direction implies a significant shear and convergence rate and, possibly, a strong vertical advection, which may have a critical impact on the communities’ distribution. Those changes in horizon speed were observed in both edges of the spiral-like feature by ADCP data during the cruise (see Figure 12 in Pinto et al., 2022). Those results possibly explain the main community composition differences on samples 53A and 56A, which were acquired near the west margin of the highly dynamic sub-mesoscale spiral-like feature (Figure 1b), which is more prone to vertical mixing in both directions. Also, the west margin was more exposed to wind blowing from the northwest (see Figure 14 from Pinto et al., 2022), which tends to increase the vertical mixing near the surface. Thus, the 53A sample inside the structure (subtropical hydrographic conditions) was possible by the influence of upward currents explained by the lower dense spiral-like feature uplift over the polar water. This shift could explain why typical DCM communities were observed in the surface sample. In contrast, the 56A sample is in the structure’s margin but within polar hydrographic conditions. Here the downward currents due to subsidence of polar water in the margin of the spiral-like structure can explain the observation of typical surface communities in typical DCM depths. In turn, this can boost ammonification and NH_4_^+^ oxidation (Azam et al., 1983; Smith and Mackenzie, 1987). Due to its dynamics, bacterial degradation is high, promoting the recycling of particulate and dissolved organic material (Ward, 2000). Raes et al. (2018) stated that this growth in the South Pacific Ocean increased the concentrations of NO_2_^–^ and NH_4_^+^ near the spiraling arm of the upwelling area. In our study, the NH_4_^+^ concentrations were below the detection limit, but it was possible to see an interesting pattern concerning the NO_2_^–^ concentration. At the surface, sample 53A, the most different surface samples in terms of prokaryotic and protists community structure, presented the highest concentration of NO_2_^–^.

The prokaryotic communities were dominated by Proteobacteria and Cyanobacteria, at a phylum level (Supplementary Figure 1), similar to other studies (e.g., Kong et al., 2021; Sunagawa et al., 2015). Considering Cyanobacteria, *Prochlorococcus* was the predominant genus, and SAR11 was the most abundant order of Proteobacteria. These organisms are capable of growing in oligotrophic waters (Sowell et al., 2009). In the NPSF, the nutrient concentrations were relatively low, and, in the case of nitrate, the values were practically absent. These low values can be a preference for the organisms that belong to the *Prochlorococcus* genus, which can prosper under low nutrient concentration areas (Techtmann et al., 2015). *Prochlorococcus* was the genus with the highest abundance in surface and DCM waters. It was possible to detect a decrease in *Prochlorococcus* abundance in sample 53A, where the nitrite values were the highest, similar to Li Y. Y. et al. (2018), where this genus was more abundant in N-deficient samples. An interesting pattern was also observed with other Cyanobacteria genus. *UCYN-A* was found in all surface samples except for sample 53A, and at DCM was only detected in sample 56A. This genus tends to be present in N-deficient samples and proliferates more in oligotrophic areas (Krupke et al., 2014). As mentioned above, it is a possibility that the sample 53A was the closest to the boundary of the spiral-like structure. Its location could explain why these genera have these distribution patterns. As for the SAR11 clades, we found resemblances with the study of West et al. (2016) in the South Pacific Ocean. *Clade Ia* presented a higher abundance in surface waters when compared to the DCM, where it was proportionally replaced by *Clade Ib*.

Regarding the protists communities, Syndiniales, a parasite from the supergroup Alveolata, was the dominant class. These results were similar to results found in other studies (e.g., Guillou et al., 2008). These organisms tend to be opportunistic and can infect several hosts, including other dinoflagellates and radiolarians (Siano et al., 2011; Bråte et al., 2012). These organisms can release dissolved organic material into the environment, due to the destruction of host cells (Lefèvre et al., 2008; Pearman et al., 2017). This leads to the production of dinospores, which gives a nutrient supply to the higher trophic levels. To evaluate how Syndiniales are influenced by the nutrients, a Spearman relationship was performed (Supplementary Figure 6) and it was demonstrated that these organisms were positively correlated with phosphates, suggesting that Syndiniales play a role in the biogeochemical cycles in the NPSF.

Protists also displayed a particular horizontal distribution across the NPSF (Supplementary Figure 2), and some genera demonstrated intriguing distribution patterns. *Nassellaria*, an important genus for the biogeochemical cycles, especially in the silica cycle, was only found in sample 53A at the surface, and in the DCM samples located in the west region (Figure 4b). It is known that this genus distribution is influenced by nutrient availability, primary productivity, and specific water mass (Liu et al., 2017). The samples where *Nassellaria* was detected presented relatively high values of the nutrients analyzed, this could justify its presence. The distribution of these organisms tends to be influenced by the concentration of nutrients, especially by the nitrogen: phosphorus ratio (Litchman and Klausmeier, 2008). However, it should be taken into consideration that the study area is an oligotrophic area, and the nutrient concentration of the samples was relatively low in comparison to other studies, so there is the possibility that the inorganic nutrients could be limiting and influence the shape of the communities’ distribution. Thompson et al. (2012) stated that UCYN-A organisms are generally found in association with eukaryotes that belong to the Prymnesiophyceae class. In our study, it is possible to find UCYN-A organisms in the same samples as the organisms of the family Chrysochromulinaceae (from the Prymnesiophyceae class). This association (Supplementary Figure 7) suggests that the nitrogen fixed by UCYN-A enters the microbial loop through the Prymnesiophyceae, which are important primary producers and mixotrophs (Zubkov and Tarran, 2008; Unrein et al., 2007; Jardillier et al., 2010; Cuvelier et al., 2010). Recent research has revealed that UCYN-A is not merely an endosymbiont but has evolved into a nitrogen-fixing organelle, termed the “nitroplast,” in certain marine algae (Coale et al., 2024). This discovery indicates a more intricate relationship between UCYN-A and its host, with implications for understanding organelle evolution and nitrogen cycling in marine ecosystems. The nitroplast has been shown to divide in synchrony with the host cell and import proteins encoded by the algal genome, characteristics typical of organelles (Coale et al., 2024). This tight integration suggests that the contribution of UCYN-A to nitrogen fixation and carbon cycling in marine environments may be even more significant than previously thought. There remains a possibility that the algal host containing the nitroplast calcifies, which could have ramifications for the contributions of N2-based new production to vertical carbon fluxes and impact the susceptibility of the host to ocean acidification (Karl et al., 2012; Doney et al., 2009).

As for the horizontal scale, certain microorganisms demonstrated a significant distance-decay relationship. Showing that geographical distance, alongside the environmental gradients, has a role in shaping microbial communities (Wu et al., 2020). In this study, *Radiolaria*, at the surface, demonstrated a significant distance-decay relationship, similar to Zhao et al. (2020). The total distance between the samples analyzed in this study was 118.92 Km, which is a relatively short distance. In studies where the distance between samples was also relatively small (e.g., Li Y. et al., 2018; Wang et al., 2020), a strong distance-decay relationship was also not found. It has been stated that the limitation of microbial dispersal intensifies with increasing geographic distances (Wu et al., 2018). Another factor that also influences communities’ capacity for dispersal is body size. Larger-bodied microorganisms exhibit significantly shorter dispersal scales compared to smaller-bodied microorganisms (Villarino et al., 2018). Other factors that are capable of influencing the patterns of distance-decay are dispersal selection, and environmental parameters (Zhou and Ning, 2017; Wu et al., 2018).

Overall, the results from this study show that the distribution patterns and factors that influence the alpha and beta diversities of both prokaryotic and protists communities in the oligotrophic waters of the subtropical North Pacific are very similar. Temperature, depth, and longitude were identified as the main factors that shaped both communities across the North Subtropical Front. The majority of the microbial communities, except for *Radiolaria* at the surface, did not demonstrate a significant distance-decay relationship, showing there is no major dispersal limitation at up to 120 Km, approximately, in the study area. However, nine prokaryotic genera (*Formosa*, *Marinoscillum*, *NS4 marine group*, *NS5 marine group*, *Urania-1B-19 marine sediment group*, *Ascidiaceihabitans*, *Roseovarius*, *Thioalkalispira* and *Candidatus nitrosopumilus*), some of which with important ecosystem functions (e.g., *Candidatus nitrosopumilus*) exhibited significant distance-decay relationships. These results emphasize the relevance of including horizontal gradients to have a better understanding of the distribution and composition of the microbial communities. However, the sub-mesoscale dynamics in the fronts can enhance vertical velocity locally, which can affect the composition and distribution of those boundary regions. Future studies should be focused on how spatiotemporal scales and nutrient concentrations impact the ecological drift of these communities.

## Data Availability

The datasets presented in this study can be found in online repositories. The names of the repository/repositories and accession number(s) can be found in this article/Supplementary material.

## Footnotes

1
https://doi.pangaea.de/10.1594/pangaea.903405

## Supplementary material

Supplementary Figure 1 Relative abundance of the phyla.

Supplementary Figure 2 Relative abundance of supergroup communities.

Supplementary Figure 3 Significative spearman correlation between *Litoricola* and temperature.

Supplementary Figure 4 Significative spearman correlation of eukaryotic genera and temperature.

Supplementary Figure 5 Significative distance-decay relationship and Bray-Curtis similarity of prokaryotic and eukaryotic genera.

Supplementary Figure 6 Significant Spearman Correlations (Dinoflagellata Class).

Supplementary Figure 7 Significative Spearman correlation between UCYN-A and chrysochromulinaceae.

Supplementary Table 1 Upstream analysis of the samples for 16S analysis.

Supplementary Table 2 Upstream analysis of the samples for 18S analysis

Supplementary Table 3 **(a)** Output of permanova analysis for the prokaryotic communities. **(b)** Output of permanova analysis for the eukaryotic communities.

Supplementary Table 4 **(a)** Spearman correlations of the prokaryotic community between the alpha diversity measures and environmental parameters. **(b)** Spearman correlations of the eukaryotic community between the alpha diversity measures and environmental parameters.
