# Supplementary material for: Horizontal distribution of marine microbial communities in the North Pacific Subtropical Front
Source: Front Microbiol. 2024 Dec 24;15:1455196. doi: 10.3389/fmicb.2024.1455196 (PMC11703956; doi:10.3389/fmicb.2024.1455196)
Supplement: Supplementary Figure 1 — Relative abundance of the phyla. [file Data_Sheet_1.zip › Data Sheet 1/Supplementary Table 1.DOCX]

| Sample ID | input | filtered | denoisedF | denoisedR | merged | nonchim | Final Output |
| --- | --- | --- | --- | --- | --- | --- | --- |
| 3A | 76148 | 63492 | 61016 | 61913 | 49108 | 46186 | 41523 |
| 4A | 126354 | 104331 | 102026 | 102506 | 81589 | 77934 | 70029 |
| 7A | 47128 | 38427 | 35064 | 35851 | 27836 | 26094 | 22628 |
| 8A | 38147 | 31181 | 28872 | 29287 | 25257 | 24023 | 22492 |
| 11A | 56201 | 46747 | 43382 | 44078 | 36613 | 34323 | 30507 |
| 12A | 42451 | 34660 | 31712 | 32198 | 26619 | 25033 | 22594 |
| 14A | 78690 | 62935 | 59656 | 60246 | 45954 | 42383 | 40809 |
| 15A | 72153 | 57706 | 52409 | 53637 | 42717 | 39996 | 34849 |
| 18A | 86160 | 70201 | 63888 | 65033 | 50679 | 47256 | 41691 |
| 20A | 56439 | 45428 | 41672 | 42354 | 33782 | 32015 | 28603 |
| 21A | 68573 | 55998 | 51977 | 52715 | 45057 | 42757 | 39107 |
| 22A | 136105 | 110629 | 105867 | 106766 | 88605 | 84444 | 76238 |
| 23A | 60317 | 49546 | 47504 | 48087 | 41227 | 39249 | 35964 |
| 24A | 167446 | 139553 | 133912 | 135124 | 118377 | 111826 | 100629 |
| 40A | 126966 | 116418 | 108681 | 109042 | 84365 | 36819 | 33952 |
| 41A | 92568 | 85908 | 81525 | 81747 | 67236 | 30915 | 28657 |
| 42A | 124453 | 114209 | 107240 | 107131 | 83249 | 36201 | 33268 |
| 43A | 59488 | 53481 | 49927 | 50367 | 38255 | 18665 | 17029 |
| 44A | 98737 | 90688 | 84401 | 84871 | 65218 | 28506 | 25872 |
| 45A | 96342 | 89315 | 84985 | 84789 | 70884 | 31605 | 29602 |
| 52A | 56738 | 45355 | 40884 | 41968 | 32875 | 30450 | 27991 |
| 53A | 112195 | 92293 | 86022 | 86992 | 68768 | 62650 | 55642 |
| 54A | 93900 | 78213 | 74936 | 75611 | 62858 | 58332 | 54808 |
| 55A | 61149 | 51104 | 49506 | 49747 | 45619 | 42991 | 41443 |
| 56A | 74972 | 60903 | 55685 | 56867 | 46219 | 42877 | 38586 |
| 57A | 73722 | 59483 | 54887 | 55781 | 47694 | 44461 | 40931 |
| 58A | 82042 | 65669 | 62527 | 63711 | 48171 | 44635 | 41010 |
| 59A | 84392 | 70827 | 67787 | 69027 | 57186 | 53502 | 49421 |
| 68A | 100888 | 92861 | 87462 | 87671 | 57401 | 24459 | 21505 |
| 69A | 70592 | 65090 | 62743 | 62690 | 54701 | 27095 | 25540 |
| 70A | 58190 | 45722 | 41873 | 42518 | 34001 | 31769 | 28006 |
| 71A | 79395 | 65049 | 60261 | 61059 | 49659 | 46061 | 43245 |
| 72A | 54326 | 44501 | 42482 | 42982 | 36857 | 34343 | 31944 |
| 73A | 63945 | 48692 | 45053 | 45781 | 38656 | 35638 | 33491 |
