# Supplementary material for: Horizontal distribution of marine microbial communities in the North Pacific Subtropical Front
Source: Front Microbiol. 2024 Dec 24;15:1455196. doi: 10.3389/fmicb.2024.1455196 (PMC11703956; doi:10.3389/fmicb.2024.1455196)
Supplement: Supplementary Figure 1 — Relative abundance of the phyla. [file Data_Sheet_1.zip › Data Sheet 1/Supplementary Table 3.DOCX]

Supplementary Table 3a: Output of permanova analysis for the prokaryotic communities.

|  | **DF** | **F. Model** | **R^2^** | **Pr (>F)** |
| --- | --- | --- | --- | --- |
| **Salinity** | 1 | 0.97665 | 0.02962 | 0.38 |
| **Temperature** | 1 | 9.0203 | 0.2199 | 0.01 |
| **Depth** | 1 | 8.981 | 0.21915 | 0.01 |
| **Latitude** | 1 | 1.015 | 0.03074 | 0.45 |
| **Longitude** | 1 | 3.8716 | 0.10793 | 0.02 |

Supplementary Table 3b: Output of permanova analysis for the eukaryotic communities.

|  | **DF** | **F. Model** | **R^2^** | **Pr (>F)** |
| --- | --- | --- | --- | --- |
| **Salinity** | 1 | 1.0296 | 0.03117 | 0.38 |
| **Temperature** | 1 | 6.0216 | 0.15837 | 0.01 |
| **Depth** | 1 | 5.9053 | 0.15579 | 0.01 |
| **Latitude** | 1 | 1.1234 | 0.03392 | 0.34 |
| **Longitude** | 1 | 3.2692 | 0.09269 | 0.01 |
