# Supplementary material for: Horizontal distribution of marine microbial communities in the North Pacific Subtropical Front
Source: Front Microbiol. 2024 Dec 24;15:1455196. doi: 10.3389/fmicb.2024.1455196 (PMC11703956; doi:10.3389/fmicb.2024.1455196)
Supplement: Supplementary Figure 1 — Relative abundance of the phyla. [file Data_Sheet_1.zip › Data Sheet 1/Supplementary Table 4.DOCX]

Table 4a: Spearman correlations of the prokaryotic community between the alpha diversity measures and environmental parameters.

| **α-Diversity** | **Environmental Parameter** | **p-value** |
| --- | --- | --- |
| *Observed* | Temperature | 0.2989 |
|  | Salinity | 0.6732 |
|  | Latitude | 0.2389 |
|  | Longitude | 0.1893 |
| *Shannon* | Temperature | 0.5791 |
|  | Salinity | 0.6373 |
|  | Latitude | 0.3774 |
|  | Longitude | 0.0235 |

Table 4b: Spearman correlations of the eukaryotic community between the alpha diversity measures and environmental parameters.

| **α-Diversity** | **Environmental Parameter** | **p-value** |
| --- | --- | --- |
| *Observed* | Temperature | 0.3025 |
|  | Salinity | 0.2153 |
|  | Latitude | 0.08125 |
|  | Longitude | 0.05358 |
| *Shannon* | Temperature | 0.7054 |
|  | Salinity | 0.2746 |
|  | Latitude | 0.0395 |
|  | Longitude | 0.1646 |
