# Supplementary figures and images for: Horizontal distribution of marine microbial communities in the North Pacific Subtropical Front
Source: Front Microbiol. 2024 Dec 24;15:1455196. doi: 10.3389/fmicb.2024.1455196 (PMC11703956; doi:10.3389/fmicb.2024.1455196)

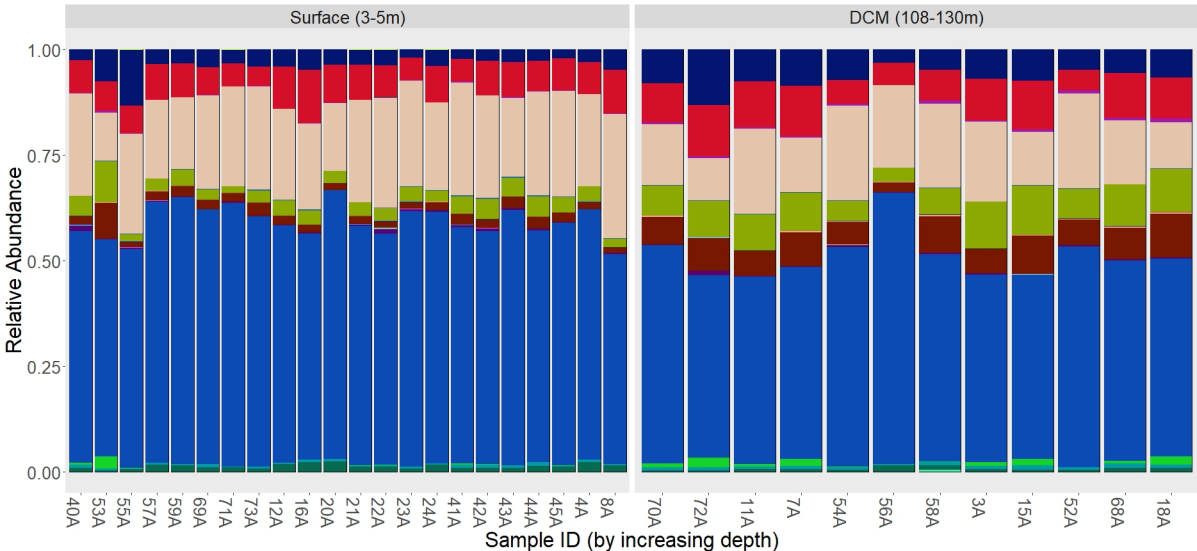

Supplement: Supplementary Figure 1 — Relative abundance of the phyla. [file Data_Sheet_1.zip › Data Sheet 1/Supplementary Figure 1.PDF]

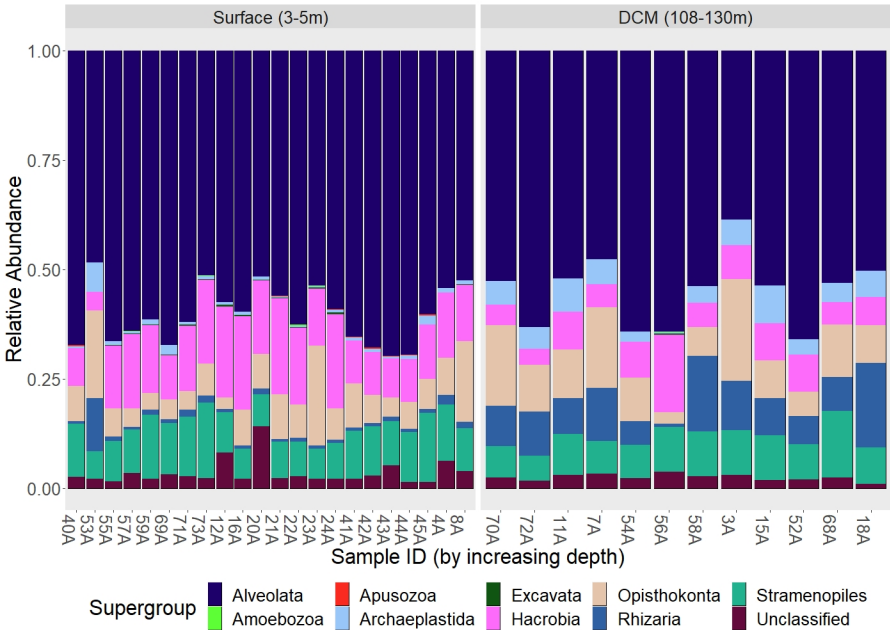

Supplement: Supplementary Figure 1 — Relative abundance of the phyla. [file Data_Sheet_1.zip › Data Sheet 1/Supplementary Figure 2.PDF]

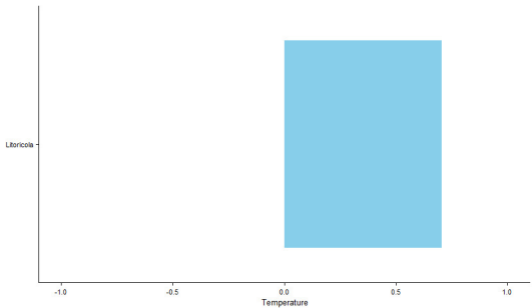

Supplement: Supplementary Figure 1 — Relative abundance of the phyla. [file Data_Sheet_1.zip › Data Sheet 1/Supplementary Figure 3.PDF]

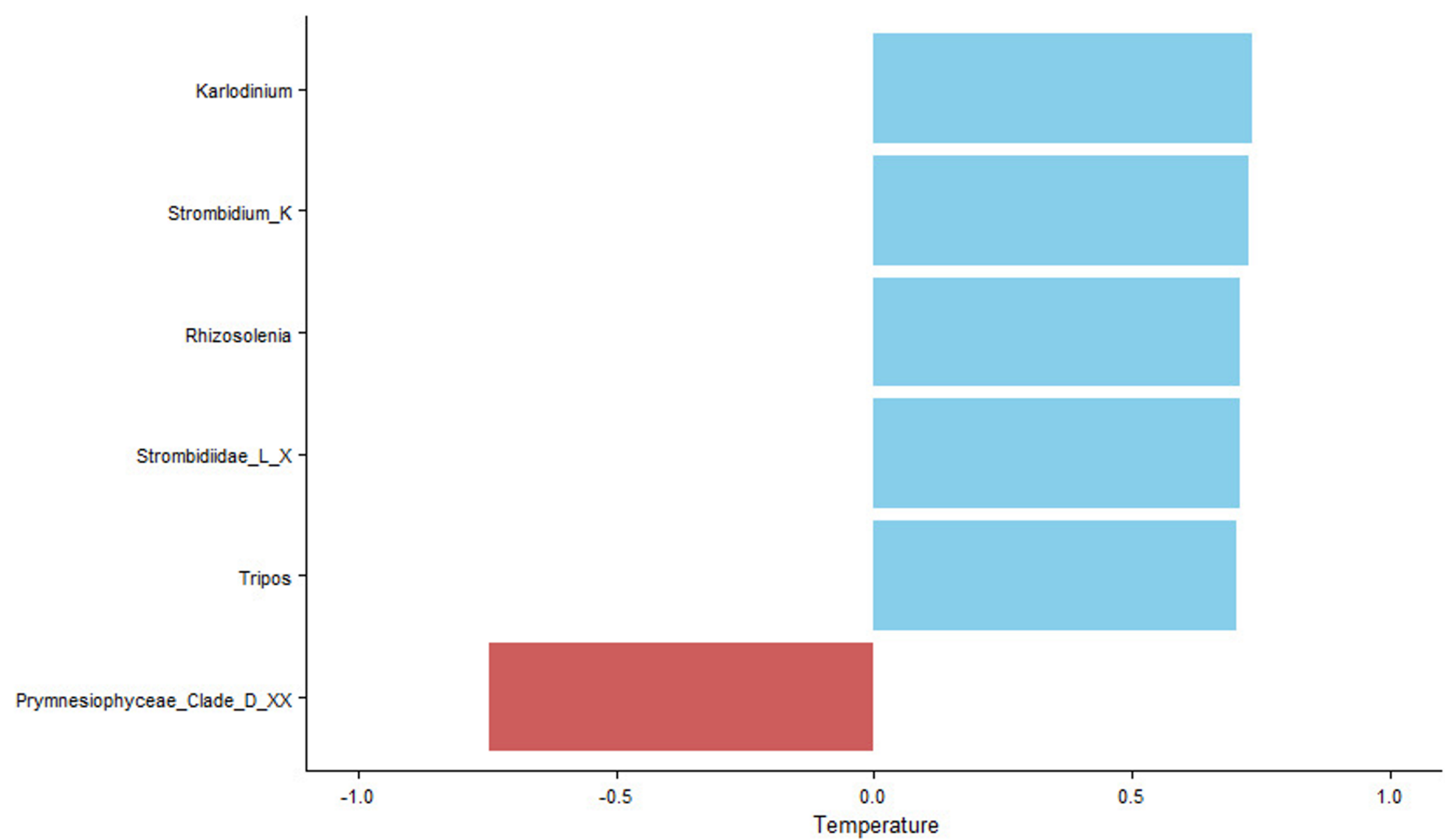

Supplement: Supplementary Figure 1 — Relative abundance of the phyla. [file Data_Sheet_1.zip › Data Sheet 1/Supplementary Figure 4.PDF]

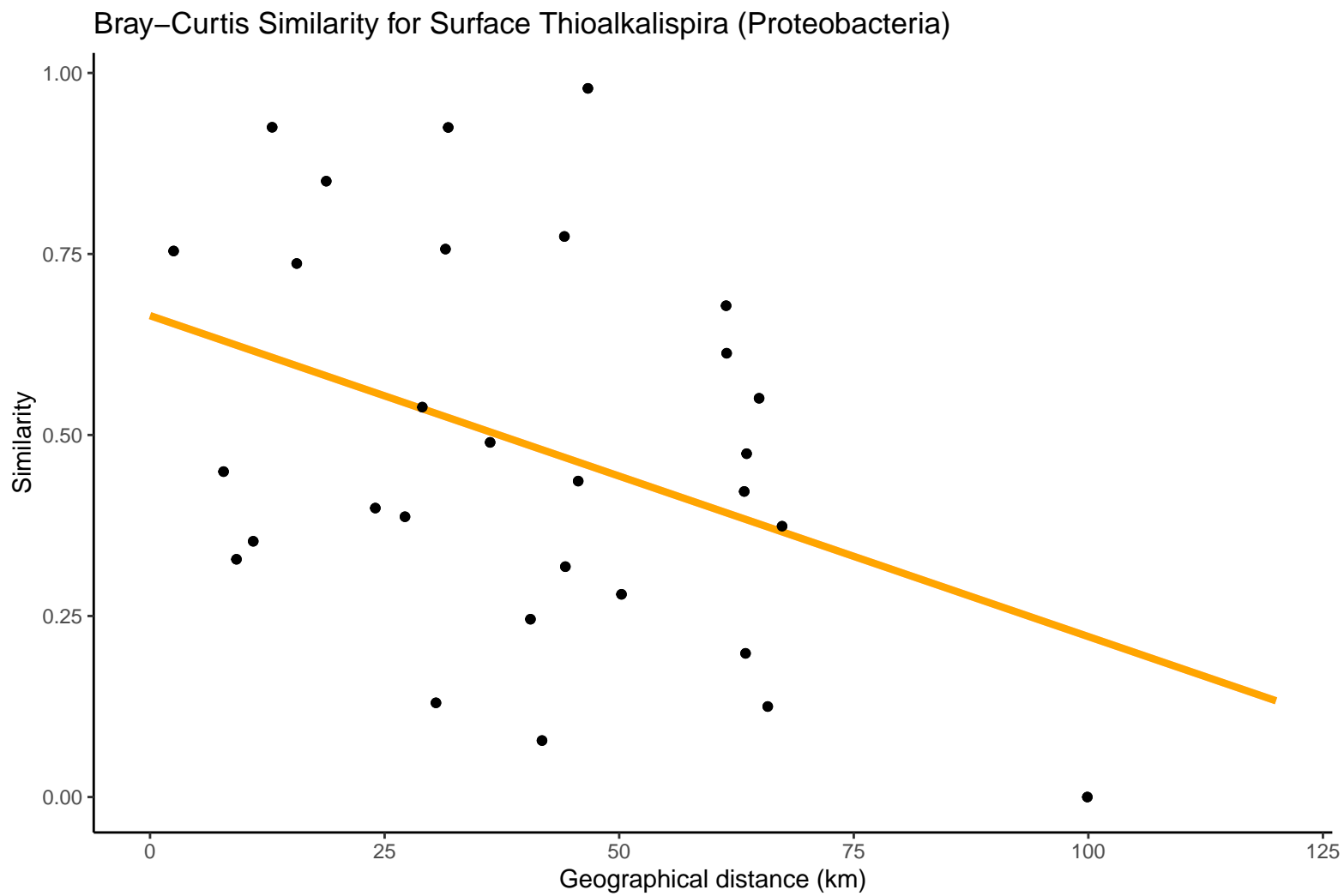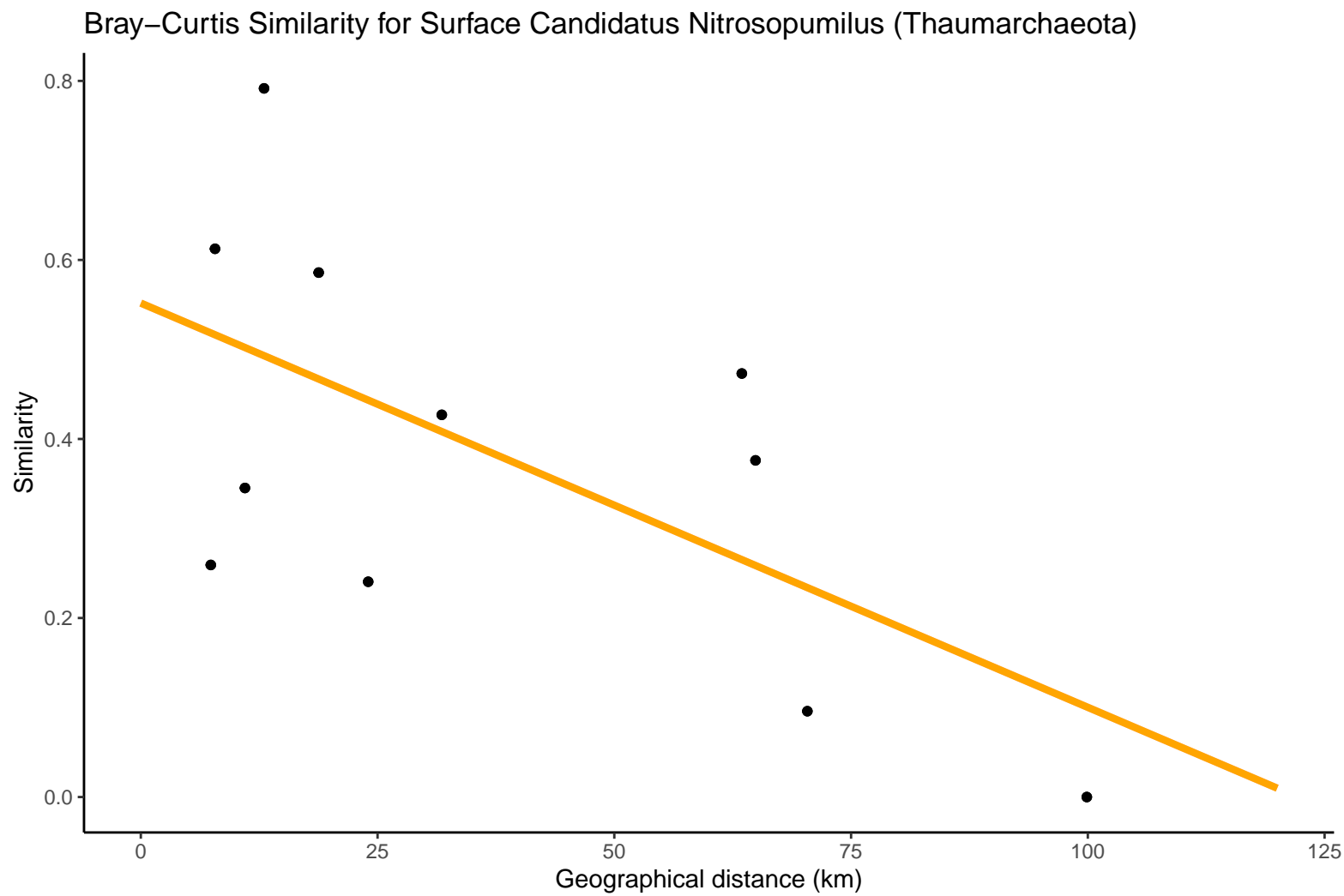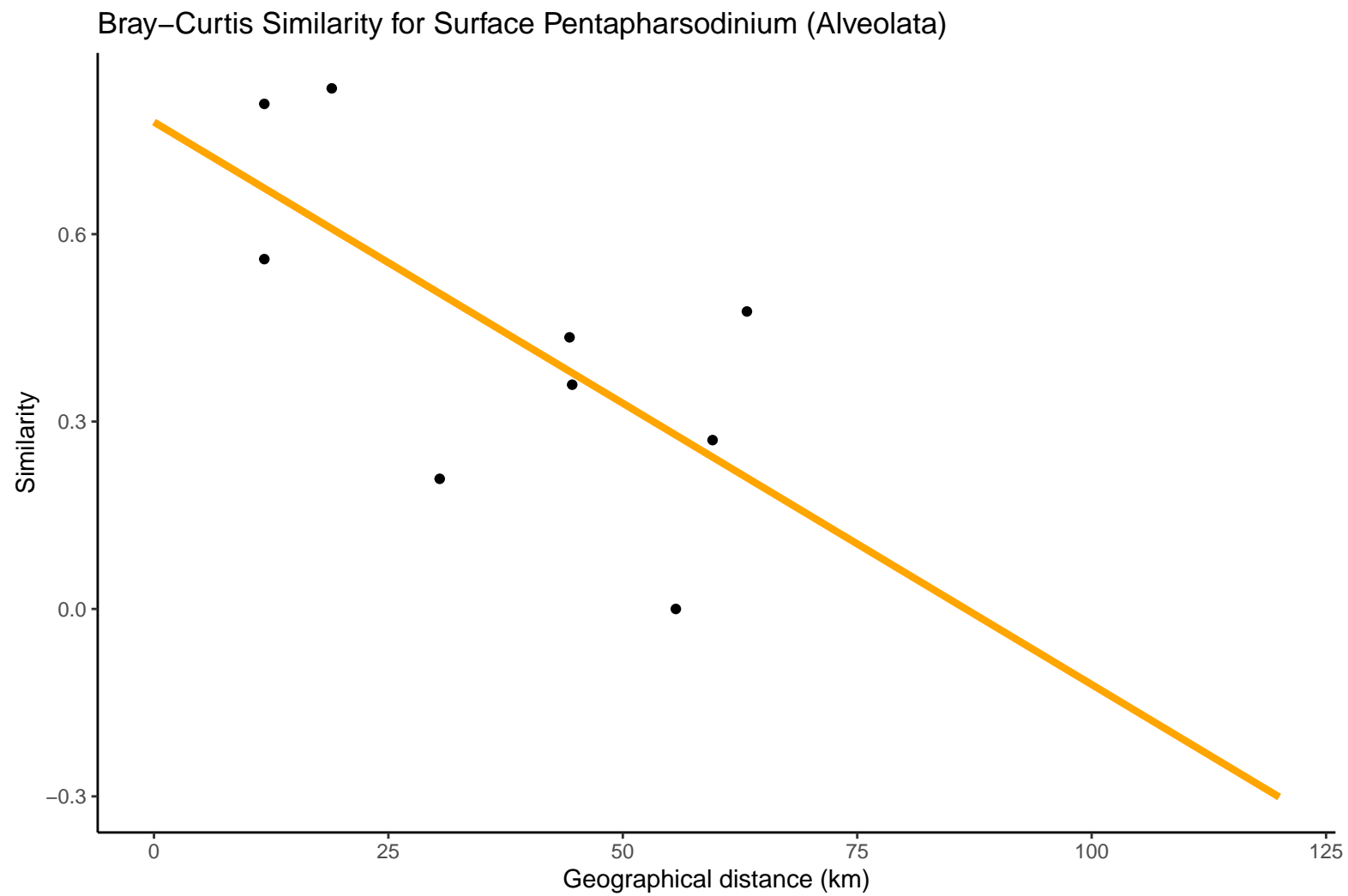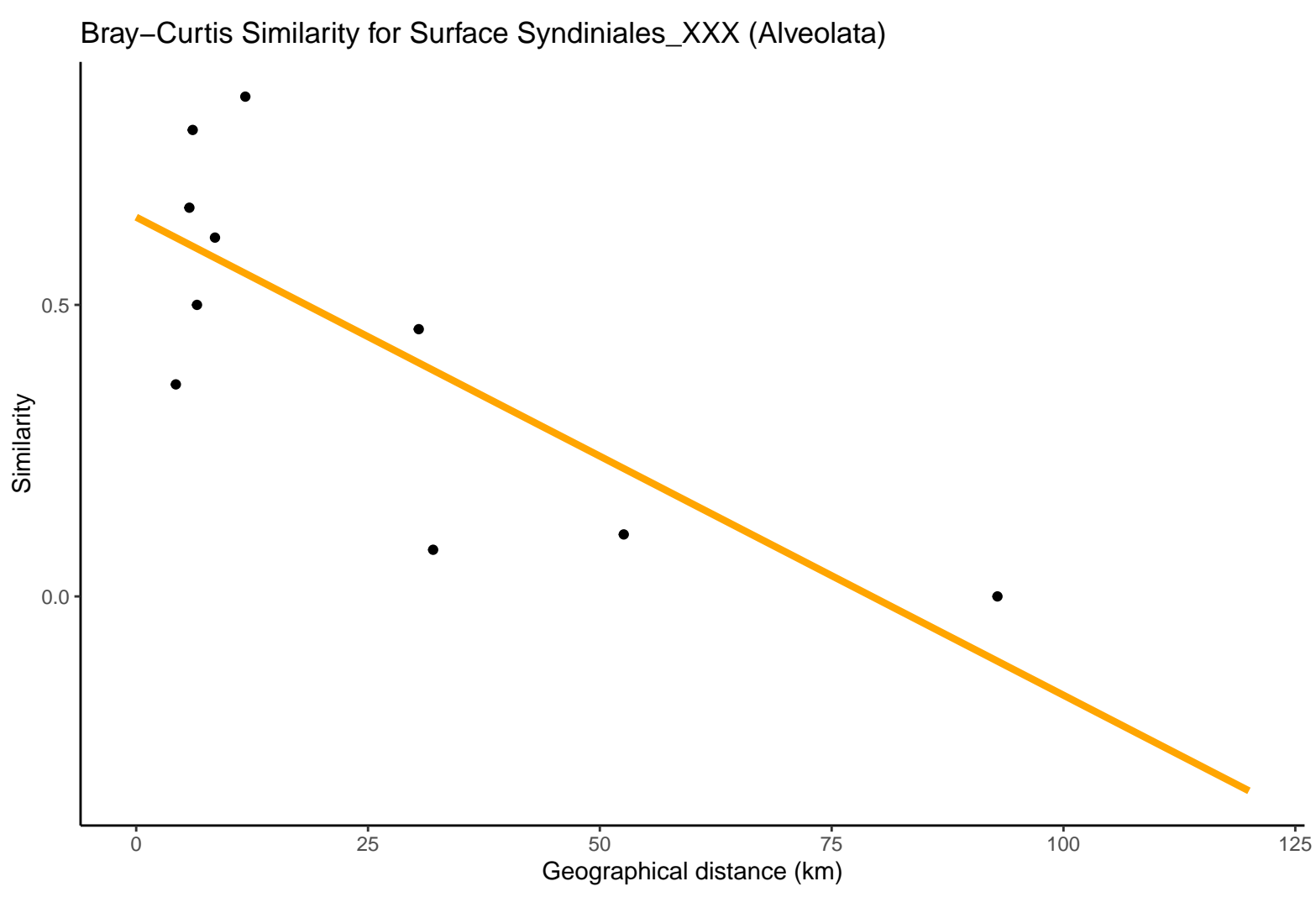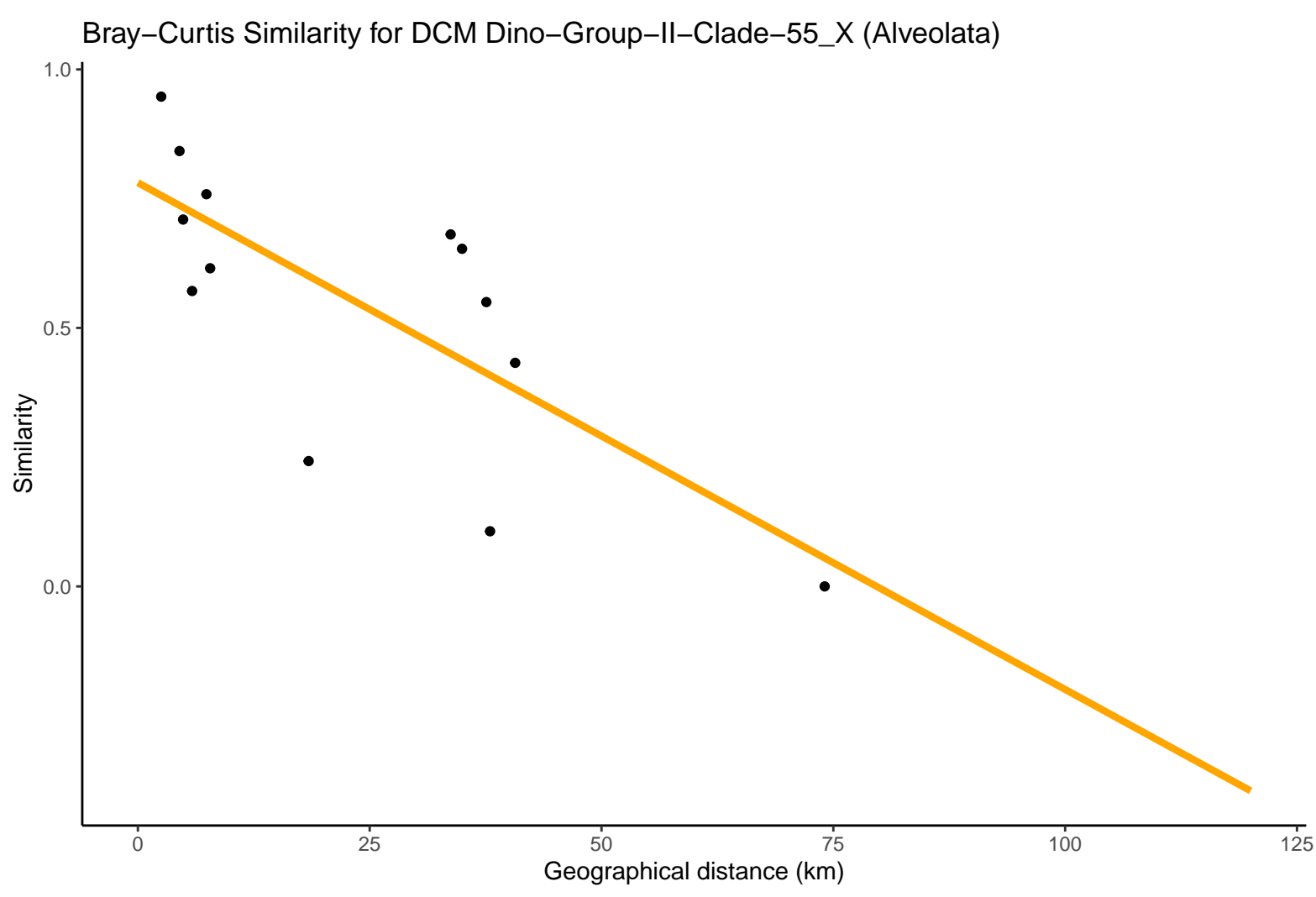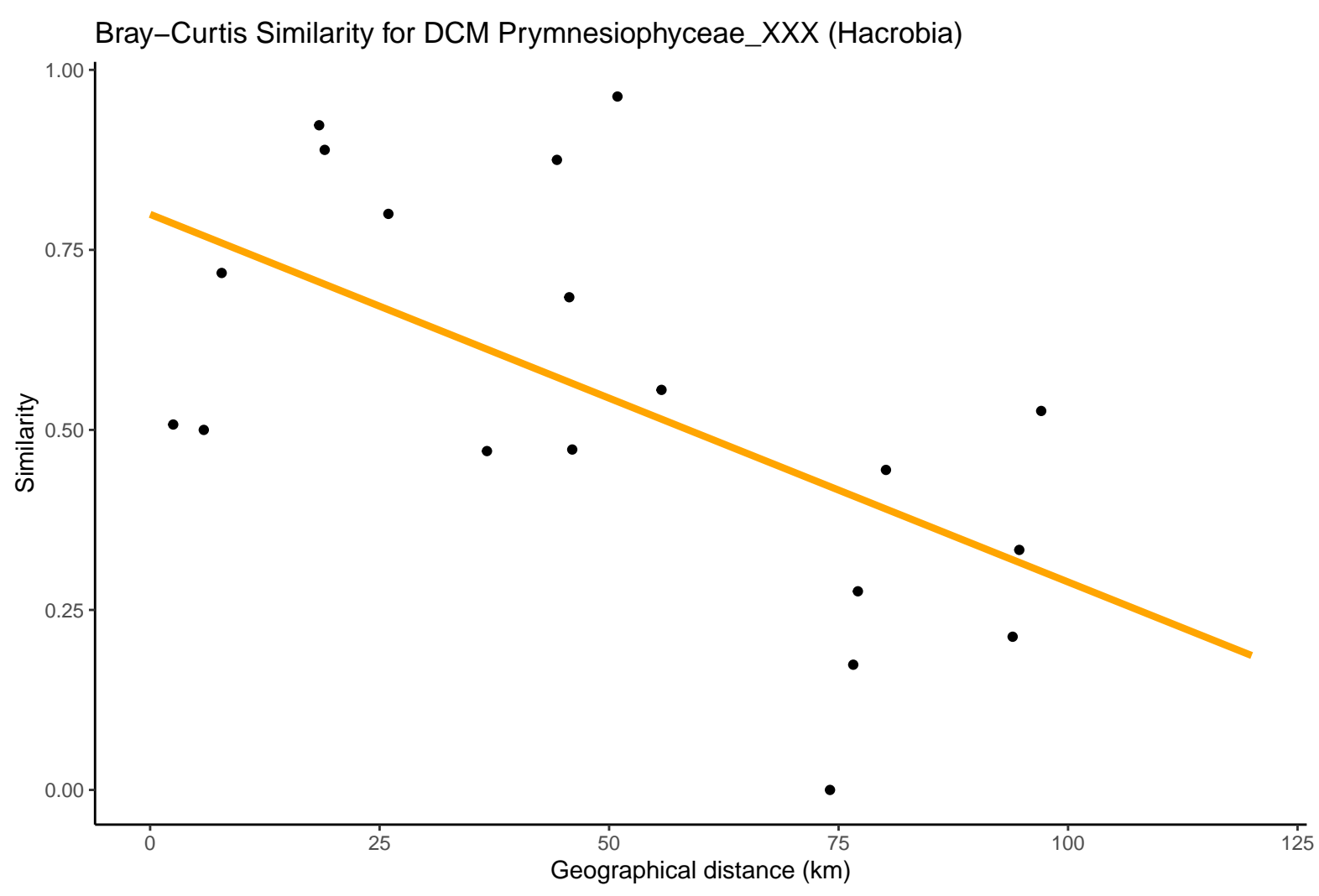

Supplement: Supplementary Figure 1 — Relative abundance of the phyla. [file Data_Sheet_1.zip › Data Sheet 1/Supplementary Figure 5.PDF]

## Significant Spearman Correlations (Dinoflagellata Class)

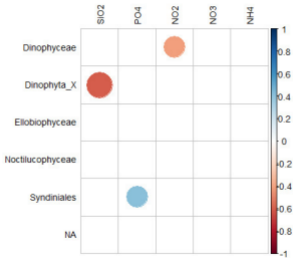

Supplement: Supplementary Figure 1 — Relative abundance of the phyla. [file Data_Sheet_1.zip › Data Sheet 1/Supplementary Figure 6.PDF]

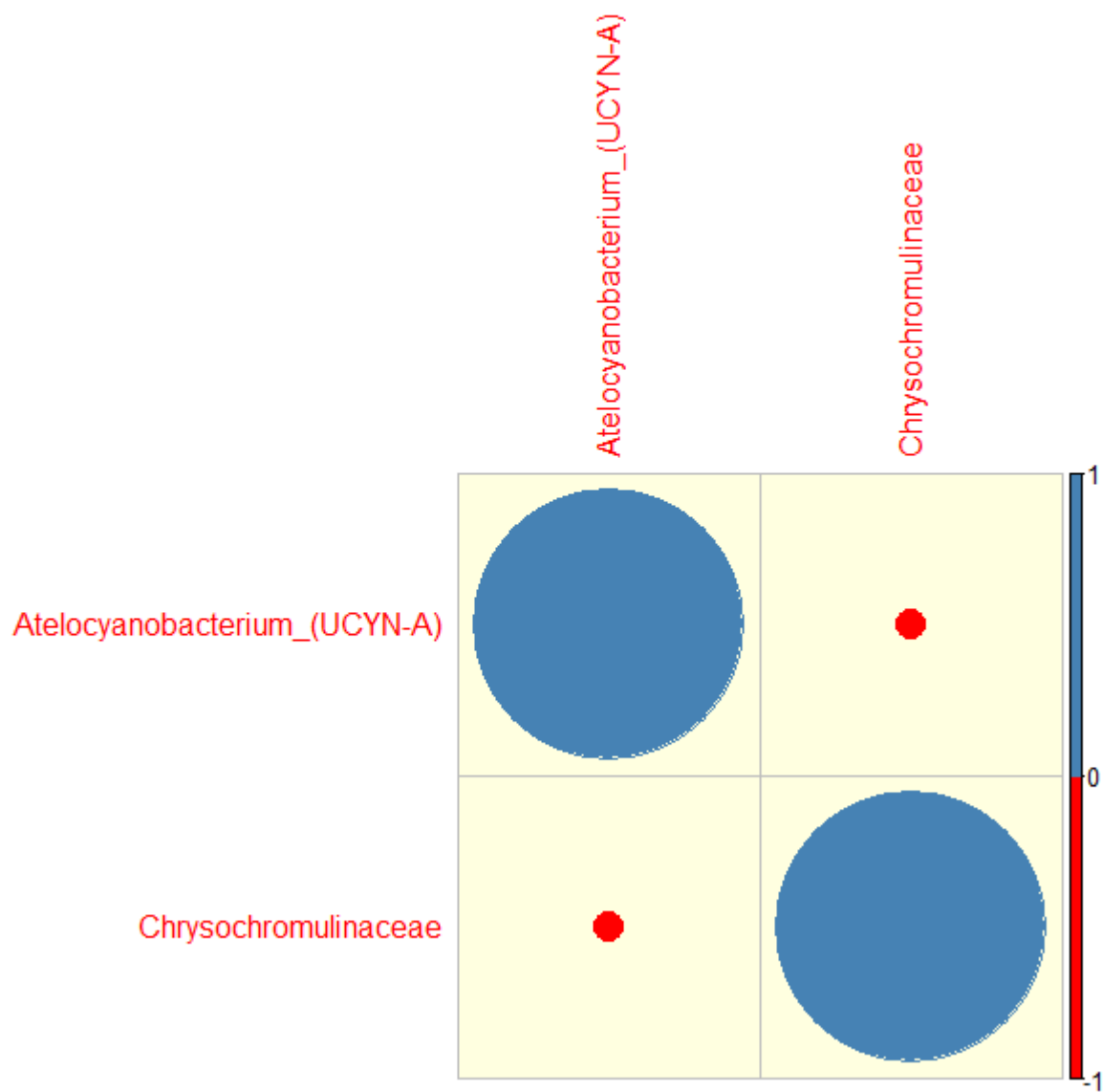

Supplement: Supplementary Figure 1 — Relative abundance of the phyla. [file Data_Sheet_1.zip › Data Sheet 1/Supplementary Figure 7.PDF]
